# Supplementary material for: Yersiniabactin-Producing E. coli Induces the Pyroptosis of Intestinal Epithelial Cells via the NLRP3 Pathway and Promotes Gut Inflammation
Source: Int J Mol Sci. 2023 Jul 14;24(14):11451. doi: 10.3390/ijms241411451 (PMC10380849; doi:10.3390/ijms241411451)
Supplement: Supplementary file 1 [file ijms-24-11451-s001.zip › ijms-2450810-supplementary.pdf]

# Supplementary Materials

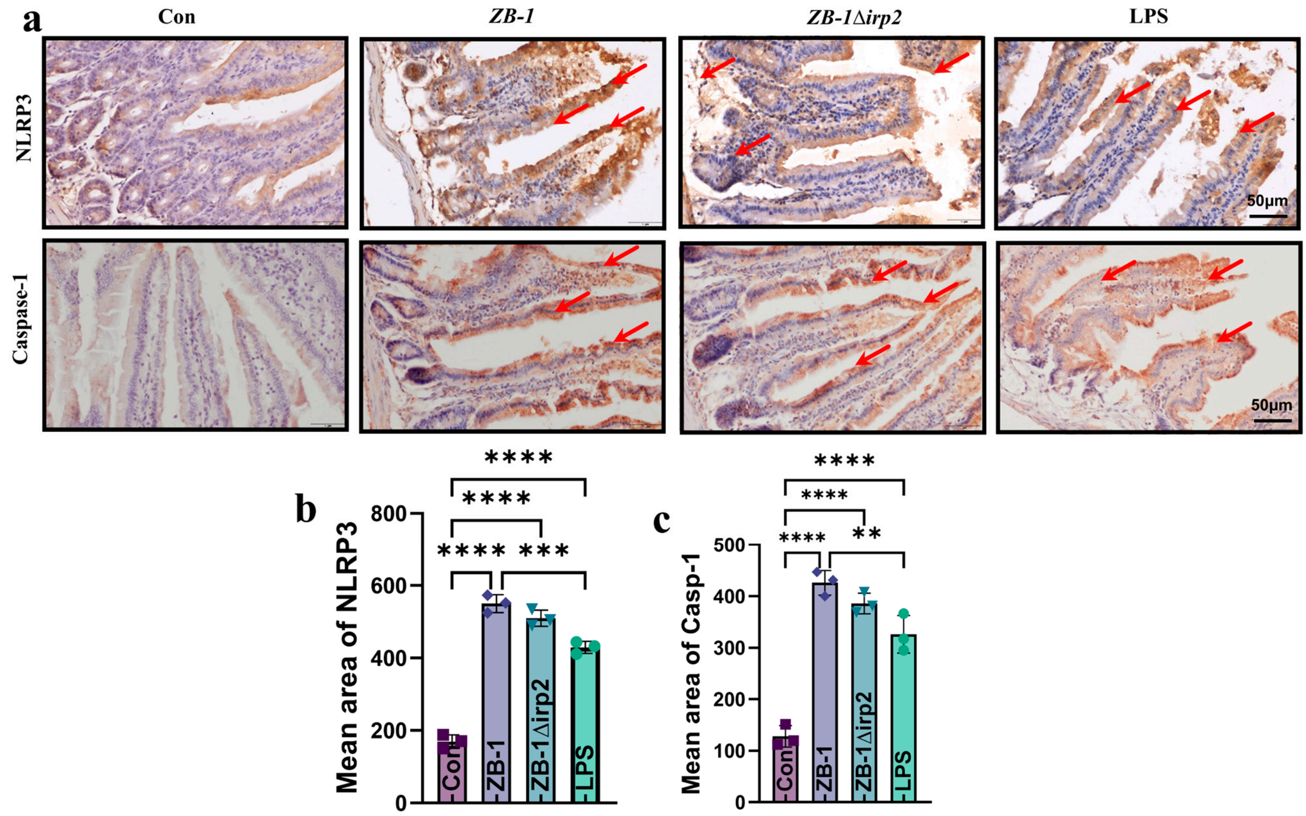


**Figure S1.** *E.coli*-Ybt induces pyroptosis via NLRP3 pathway. (**a**).Representative photomicrographs of immunohistochemistry results showing the staining of NLRP3 and Caspase-1 in different groups (Scale bar, 50 μm, typical injuries are depicted by the arrows). (**b**, **c**). The intestine' mean density of NLRP3 and Caspase-1 was determined ($n$ =3). All data are shown as the mean ± SD. **** $p < 0.0001$, and ** $p < 0.01$,.

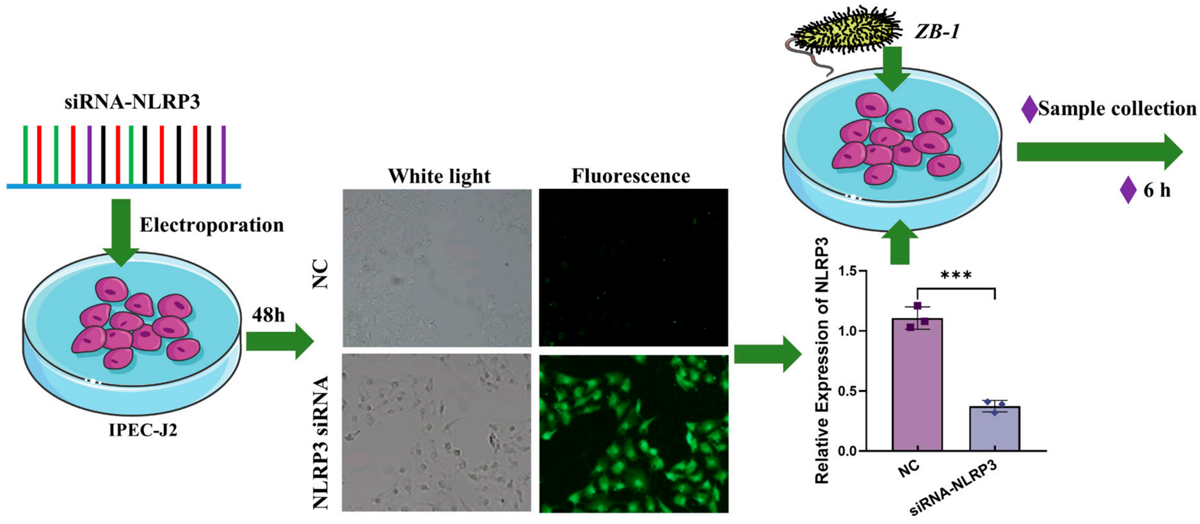


**Figure S2.** Specific siRNA was used to knockdown NLRP3. All data are shown as the mean ± SD, ($n$ = 3). *** $p < 0.001$.

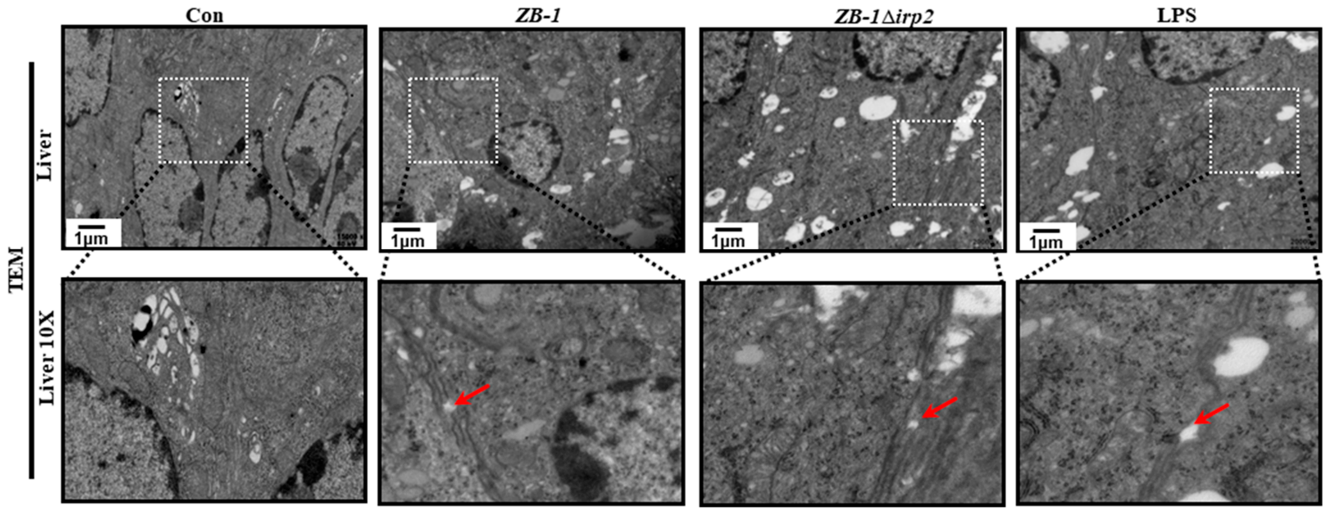


**Figure S3.** Cell membrane pore formation observed by TEM (Red arrows represent cell membrane pores; the local zoom location is indicated by the white square box).

**Table S1.** Docking score of Ybt interaction with NLRP3

| CurPocket ID | Vina score | Cavity volume (Å3) | Center (x, y, z) | Docking size (x, y, z) |
|---|---|---|---|---|
| C1 | -9.1 | 6062 | 208, 168, 108 | 26, 35, 33 |
| C2 | -8.2 | 3059 | 220, 161, 113 | 26, 26, 26 |
| C3 | -7.0 | 328 | 231, 156, 97 | 26, 26, 26 |
| C4 | -6.9 | 321 | 231, 178, 120 | 26, 26, 26 |
| C5 | -6.0 | 314 | 204, 150, 119 | 26, 26, 26 |

**Table S2.** Drug information and Vina score

| ID | Name | Formula | CAS | Vina score | MW | Molecular Structure |
|---|---|---|---|---|---|---|
| DB01396 | Digitoxin | $C_{41}H_{64}O_{13}$ | 71-63-6 | -10.7 | 764.9391 | 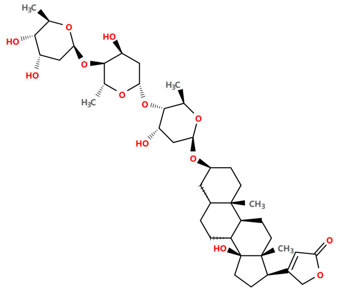 |
| DB06210 | Eltrombopag | $C_{25}H_{22}N_4O_4$ | 496775-61-2 | -9.8 | 442.4666 | 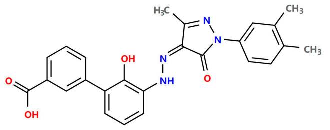 |
| DB06636 | Isavuconazonium | $C_{35}H_{35}F_2N_8O_5S$ | 742049-41-8 | -9.3 | 717.77 | 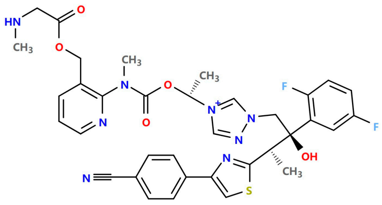 |
| DB14703 | Dexamethasone metasulfobenzoate | $C_{29}H_{33}FO_9S$ | 16978-57-7 | -9.0 | 576.63 | 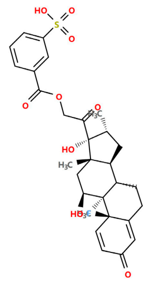 |
| DB01267 | Paliperidone | $C_{23}H_{27}FN_4O_3$ | 144598-75-4 | -8.7 | 426.4839 | 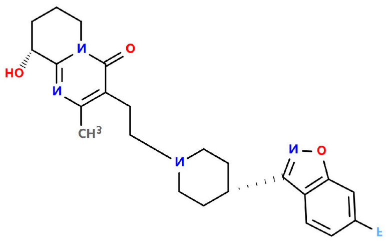 |

**Table S3**. Primers for qPCR analysis to detect mRNA levels in mouse tissues and cells

| **Name** | **Sequences** |
|---|---|
| **Primers for qPCR analysis to detect mRNA levels in mice** | |
| *β-actin* | CCTGCGGCATTCACGAAACTAC |
| XM_005887322.2 | ACTCCTGCTTGCTGATCCACAATC |
| *NLRP3* | AACTGCAGCATCTCCTGGAC |
| KF032621.1 | ACACAATCCAGCAGACCAGG |
| *ASC* | AACCCAAGCAAGATGCGGAAG |
| AB059327.1 | TTAGGGCCTGGAGGAGCAAG |
| *Caspase-1* | AAAAGTGAGCCCCTGACAGG |
| BC008152.1 | AATGAAAGACGGCACACCCA |
| *IL-1β* | AATGAAAGACGGCACACCCA |
| NM_008361.4 | GGAAGACAGGCTTGTGCTCT |
| *IL-18* | GGCTGCCATGTCAGAAGACT |
| BC024384.1 | CCTCGAACACAGGCTGTCTT |
| *GSDMD* | CAGATGGGATTGATGAGGAG |
| BC029813.1 | AATCTTTCCGATGTTCACCA |
| **Primers for qPCR analysis to detect mRNA levels in cells** | |
| *β-actin* | TGCGGGACATCAAGGAGA |
| AY550069.1 | AGGAAGGAGGGCGGAAGAG |
| *NLRP3* | GCAGCCAGTGAGCAGAG |
| JQ219660.1 | TCAACTACTACCTGGAGGAG |
| *ASC* | GTTTGTTGTCTGCTTTCAGG |
| AB873106.1 | TCAACTACTACCTGGAGGAG |
| *Caspase-1* | ACATCTGGGACTTCTTCG |
| NM_214162.1 | GCCTTGCCCTCATAATCT |
| *IL-1β* | GCAGTGGAGAAGCCGATGA |
| NM_214055.1 | GGTGGAGAGCCTTCAGCAT |
| *IL-18* | GATGGTTACTGCCAGACCTCTA |
| AY450287.1 | GGATATGCCTGATTCTGACTGTT( |
| *GSDMD* | CCCCTTCTACTTCCATGACACT |
| AK394823.1 | CCTCCGTCACCACGAACAC |
